# The role of transparency cues in afterimage color perception

**DOI:** 10.1038/s41598-017-09186-1

**Published:** 2017-08-23

**Authors:** Zhi Xiang On, Jeroen J. A. van Boxtel

**Affiliations:** School of Psychological Sciences and Monash Institute of Cognitive and Clinical Neurosciences, Monash University, Clayton, 3800 Vic Australia

## Abstract

Recent evidence has shown that afterimage perception and completion are amenable to contextual information. It has previously been shown that placing an outline around part of the afterimage can induce colors in areas that were uncolored. A thorough explanation of this effect is lacking, although this color completion was thought to be due to a diffusion-like filling-in of the uncolored patch with colors of the surrounding areas. Here, we show that an important step in visual completion is the decomposition of the visual scene into different depth layers, i.e. scission, which, we show, is guided by transparency cues in the van Lier *et al*. study. In three experiments, we show that when decomposition is prevented, color completion does not occur. We also show that this decomposition can induce color completion in real images. These results demonstrate that transparency information plays an important role in determining visual color completion processes.

## Introduction

In order to make sense of the visual world, the visual system must decompose the incoming visual information into different objects, each with their own visual features, such as shape, color, reflectance, etc^1^. How the decomposition, and reconstruction of unseen (e.g., occluded) elements takes place crucially determines our visual perception.

Scene decomposition is often thought to originate at the boundaries of objects, which determine occlusion relationships^2^, and the appearance of surface properties^3^. A clear example of the influence of boundaries on surface perception is the case of afterimages. Afterimages are rare in everyday settings, because once the eyes move, the afterimage does not line up with any physical boundary^4^. However, afterimages become very vivid once boundaries are present^4–6^. Thus, afterimages provide an important tool in investigating the influence of scene composition on surface representations.

**Figure 1:**
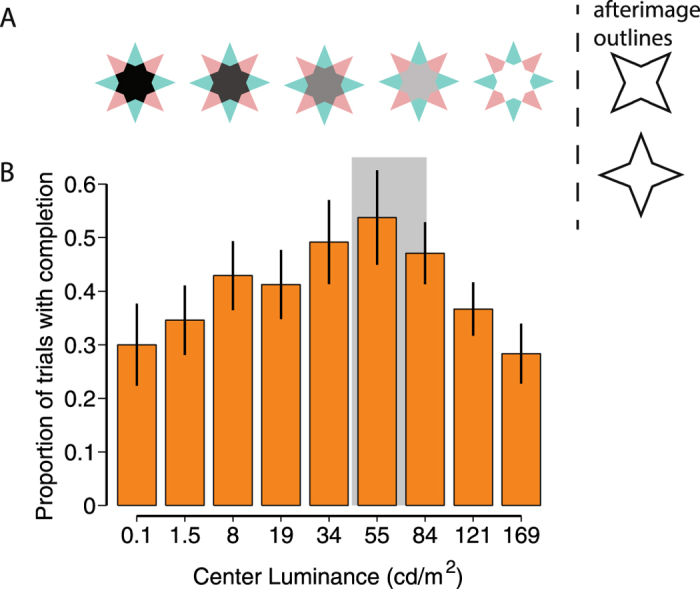
(**A**) Example stimuli, with on the right the two outlines that were shown during the time the afterimage was visible. (**B**) Mean (± s.e.m. over subjects) proportion of trials with afterimage completion dependent on the luminance level of the star**’**s center. The gray shaded area corresponds to the values that are approximately consistent with transparency.

A particularly striking example of the influence of boundaries was provided by van Lier *et al*.^5^. They created afterimages with a star-shaped stimulus that consisted of red and green points and a gray center (Fig. 1, second star from the right). When they outlined only the afterimage created by the red (or green) points, together with the center patch, only the afterimage of those points was perceived, and – surprisingly – the central patch was also filled-in with the colors of the outlined points. This processed will be referred to as afterimage completion. Afterimage completion occurred even though the center had not received a colored stimulus. It has been suggested that this filling-in occurs because the color spreads in from the points to the colorless center, and thereby averages over the entire outlined area^5, 6^. This interpretation implicitly assumes that the central patch takes little role in the processes that determine its colored appearance. Contrary to this assumption, the central patch (or more precisely, the boundary between the central patch and the points) is not devoid of scene information, as it provides important transparency cues^7, 8^. These transparency cues describe a layered representation of the visual scene in which the central patch is represented in two different colored layers: one red and one green, overlaid on top of each other. With this representation of the visual stimuli, the central patch can take a central role in the process of completion by providing the transparency cues that allow for a layered scene decomposition. If the completion of the afterimage is indeed dependent on the layered scene decomposition, one should not expect completion when transparency rules are violated. In this report, we show that transparency-induced scission – breaking the scene into different depth layers – determines whether an artificially introduced boundary can induce perceptual completion in the van Lier *et al*. stimulus, and thus we show that that scission plays an important role in determining the conscious perception of afterimages, and by extension, of any layered visual scene.

## Results

### Experiment 1: Transparency modulated by central patch luminance

In Experiment 1 we tested whether changing the luminance of the central part of the star impacts the completion of afterimages. We use the stimulus introduced by van Lier *et al*.^5^, and vary the luminance of the central patch (see Fig. 1A). Transparency rules dictate that transparency is only perceived for intermediate gray levels, and not for luminance values near black and white^9^. Specifically, assuming that the green and red stars are due to colored objects (or filters) with a certain uniform transmittance, transparency is a possible interpretation of the visual scene only when the center patch has a luminance less than the points of the stars because transmittance values less than (the maximum) 1 can only decrease overall luminance as light passes through the object. Therefore, transparency is induced when the center patch has a luminance lower than the star’s points, which in our stimuli is around 84 cd/m^2^. There is also a lower bound to transparency, assuming that the transmittance is uniform across the objects. The transmittance of the star (T_star_) is described by T_star_ = L_point_/L_background_. Therefore, the minimum luminance of the center equals T_star_ times the luminance of the points: L_center_ = T_star_ * L_point_ (ref. 9). In our stimuli this value corresponds to 41.7 cd/m^2^. These limits are indicated in Fig. 1 with the shaded gray area. If transparency cues are essential in the visual completion of color, then we should expect that maximum completion be reached between these two limits, thus we expect a quadratic (inverted-U) relationship between (log) luminance and afterimage filling-in.

Indeed, when we tested this prediction, the mean proportion of trials with afterimage completion depended on the luminance level of the central patch (Fig. 1), describing an inverted-U shape. The luminance level of 55 cd/m^2^ has the highest proportion of trials with afterimage filling-in (mean (M) ± s.e.m, M = 0.54 ± 0.09); while the conditions with a luminance of 0.1 cd/m^2^ and 169 cd/m^2^ had the lowest afterimages completion ratio (M = 0.30 ± 0.08 and M = 0.28 ± 0.06 respectively). We tested for the quadratic relationship between the center luminance and the proportion of afterimage completion trials with a linear-mixed model, comparing models with and without the quadratic dependence (see methods). We found that the quadratic dependence on (log) luminance was significant (χ^2^(1) = 57.15, p = 4.0e-14). (Earlier pilot data also revealed this pattern: χ^2^(1) = 37.42, p = 9.55e-10).

Importantly, peak afterimage perception was observed when the center patch had a luminance of 55 cd/m^2^, which is in the parameter region where luminance transparency rules are obeyed, and between the two luminance bounds that admit a transparency interpretation. The linear-mixed model peaks at 35.87 cd/m^2^ (38.75 cd/m^2^ in the pilot data), which is also smaller than 84 cd/m^2^. Furthermore, the average proportion of completion is higher for the luminance conditions of 34 cd/m2 and 55 cd/m2, than the average of 121 cd/m^2^ and 169 cd/m^2^ (one-sided t-test: *t*(11) = 2.18, *p* = 0.026, Cohen’s d = 0.63). Similar results are obtained when comparing 34 to 121 cd/m^2^ (*t*(11) = 1.95, *p* = 0.039, Cohen’s d = 0.56), and 55 to 169 cd/m^2^ (*t*(11) = 2.15, *p* = 0.028, Cohen’s d = 0.62). For the pilot data these values were: *t*(17) = 2.46, *p* = 0.012, Cohen’s d = 0.58; *t*(17) = 2.40, *p* = 0.014, Cohen’s d = 0.57; and *t*(17) = 1.96, *p* = 0.033, Cohen’s d = 0.46.

Outside the region consistent with transparency, the proportion of trials with visually-completed afterimages decreased. However, there is a significant proportion of completion, even at the lowest (*t*(11) = 3.92, *p* = 0.0024, Cohen’s d = 1.13) and highest (*t*(11) = 5.04, *p* = 0.00038, Cohen’s d = 1.46) luminance values (pilot data: *t*(17) = 3.81, *p* = 0.0014, Cohen’s d = 0.90; *t*(17) = 5.28, *p* = 6.18e-05, Cohen’s d = 1.24). Because transparency rules would predict no completion in these conditions, these findings could indicate that there is a different process operating in addition to the transparency cues. It is important to realize, however, that proportions are bounded by 0 and 1, and thus all proportions are by necessity 0 or larger, and thus finding significantly positive proportions is not that surprising. Importantly, most participants had small proportions of completion for 0.1 cd/m2 and 169 cd/m^2^, clustering close to 0, with the mean driven up by a few participants. On the other hand, the proportions were clearly positive for e.g. 55 cd/m2 and 84 cd/m^2^. To estimate the most likely reported proportion (i.e. mode) from our sample, we fitted beta functions (with betafit in MATLAB®) to the obtained proportions. We averaged the proportions of 0.1 and 169 cd/m^2^ per participant, to which the beta function was then fit. The mode was 0.12 (pilot data: 0.12), which is considerably smaller than the observed mean of 0.3 in Fig. 1. The mode was 0.51 for the data averaged over conditions 55 cd/m2 and 84 cd/m^2^. These data suggest that, while there is scope for small additional processes (including color diffusion and color induction^5^, attentional lapses, finger errors), a large contribution to the data in Fig. 1 is the presence of transparency cues.

Finally, we compared two models that can be proposed to describe our data (see methods for details). The first model is based on the transparency rules, assuming maximal afterimage completion occurs over the region consistent with transparency (between 41.7 and 84 cd/m^2^), and decreases on either side of it. The second model is based on assuming the completion is maximal at equiluminance (at 84 cd/m^2^), and decreases on either side of equiluminance. Both models have 3 free parameters: the maximum proportion of completion, and the 2 slopes on either sides of maximum completion. Models were compared with Bayesian information criterion (BIC) measures^10, 11^, and posterior probabilities^12, 13^. We found that the transparency model had a BIC of −38.67, while the equiluminance model had a BIC of −32.46, which is a difference of 6.21, which qualifies as ‘strong’ evidence for the transparency model^11^. Posterior probabilities (‘Schwarz weights’^13^) were 0.957 for the transparency model, and just 0.043 for the equiluminance model. Thus, the transparency model is >22 times (i.e., 0.957/0.043) more likely than the equiluminance model.

### Experiment 2: Same luminance edges, but different transparency

Experiment 1 showed that when luminance cues were consistent with transparency rules, the afterimage completed within the introduced boundaries. When the transparency rules were violated, there was a reduced amount of completion. One may argue that this lack of completion was not due to the violation of transparency rules, but instead due to the introduction of sharp luminance boundaries between the star’s points and the center, which become stronger as the luminance difference between the center and the points increases. This effect could potentially have prevented the spread of the afterimage color. However, this explanation cannot explain why the peak proportion of afterimages does not occur at the luminance of the star’s points (at 84 cd/m^2^), but at a lower luminance, and is not supported by our model comparison in experiment 1. An explanation in terms of transparency rules, however predicts the maximum to occur at or below the luminance of the star’s points (see gray area in Fig. 1), consistent with our findings.

**Figure 2:**
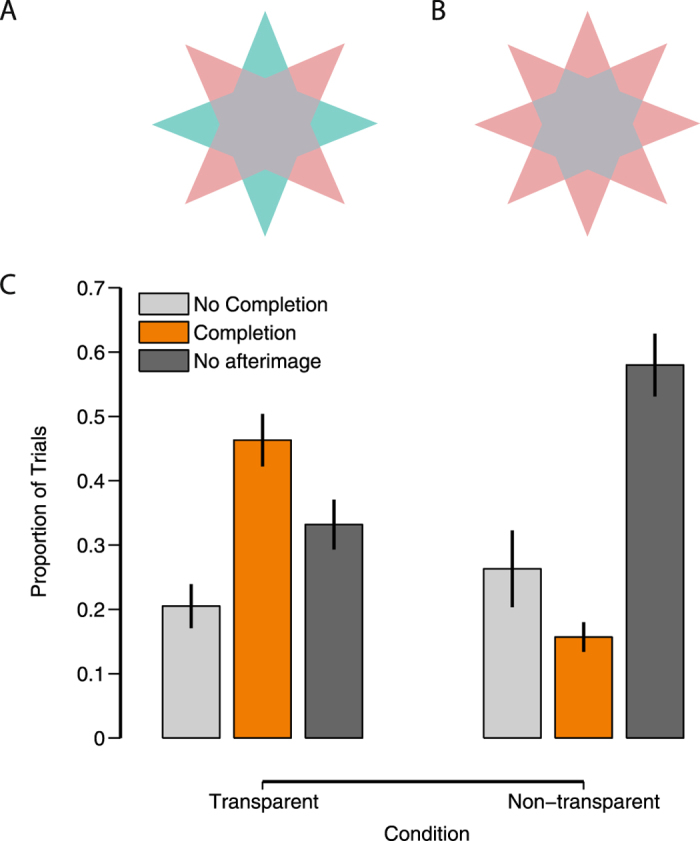
Completion dependent on transparency. (**A**) Example stimulus consistent with transparency interpretation, (**B**) example stimulus not consistent with transparency, (**C**) Response distributions dependent on whether the stimulus is consistent or inconsistent with transparency. Error bars represent s.e.m. over subjects.

Nevertheless, we conducted another experiment to empirically test the hypothesis that, at a given luminance boundary between the star’s center and points, transparency cues can determine whether afterimage completion occurs. In this experiment, we used the same star-shaped stimuli. In some conditions the arms of the star were green and red (or blue and yellow), as before (see Fig. 2A). In other conditions all arms were the same color (see Fig. 2B for an example). The second condition creates the same luminance boundaries as the original stimulus, and is in that respect identical to the original stimulus. However, it lacks a transparent percept (see Fig. 2B). As in experiment 1, we measured whether or not the center of the afterimage was completed. We predicted less afterimage completion for the second condition (without transparent percept).

We found (Fig. 2C) significantly more perceptual completion in the transparent condition (M = 0.46 ± 0.04), than in the non-transparent condition (M = 0.16 ± 0.02): two-tailed paired *t*(19) = 6.70, *p* < 0.0001, Cohen’s d = 1.50. The mean difference in afterimages was 0.31 with a 95% confidence interval ranging from 0.22 to 0.40. There was also a significant decrease in the number of trials without an afterimage (*t*(19) = 6.10, *p* < 0.0001, Cohen’s d = 1.37), but no significant change in the no-completion trials (*t*(19) = 1.21, *p* = 0.24, Cohen’s d = 0.27). These results show that, while keeping luminance discontinuities the same, when color configurations induce a transparent percept of the inducer, afterimage completion is high. Completion is low when a transparent percept is prevented. Using the beta function fitting procedure as in experiment 1, we obtain a mode of 0.085 in the latter case, showing that afterimage completion is, on the whole, minimal when transparency cues are absent.

### Experiment 3: Correlation between transparency perception, and afterimage completion

To further quantify the link between transparency and afterimage completion, we asked participants to rate all the inducer stimuli shown in the previous two experiments in terms of whether they appeared transparent. The rate of perceived transparency was then correlated to the rate of completion in the previous two experiments. This allowed us to determine whether transparency correlated with completion.

**Figure 3:**
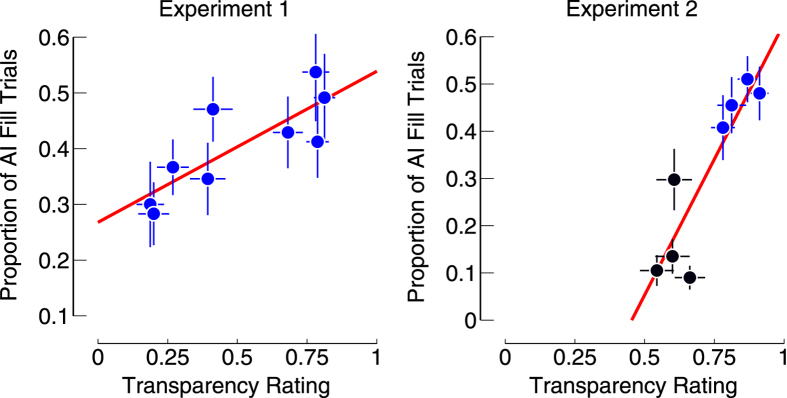
Correlation between transparency ratings and afterimage completion. Each dot represents one of the conditions tested in each experiment, and is the mean (± s.e.m.) over subjects for both the transparency rating, and the afterimage completion. For experiment 2, the blue dots show the data for the conditions similar to Fig. 2A, and black dots show the data for the conditions similar to Fig. 2B.

Both experiments showed a significant correlation between perceived transparency and afterimage completion (Exp. 1: *r* = 0.82, *p* = 0.007; Exp. 2: *r* = 0.89, *p* = 0.003), showing that there was a strong correlation between visual completion and the perception of transparency (Fig. 3).

## Discussion

Here we have shown that afterimage strength depends not only on the presence of boundary contours, as shown previously^4–6^, but that transparency cues determine to a large extent whether afterimages are perceptually completed. The transparency cues induce scission (the segmentation of a scene in multiple depth layers), and our findings suggest that this scission is an important precondition in the visual completion processes, adding to its importance for visual perception, where it has been suggested to also influence color contrast^14^, neon-color spreading^15^, and the watercolor illusion^16^. We note that in other situations scission may be induced by other cues, such as occlusion, or binocular disparity, but in the stimuli by van Lier *et al*. transparency appears to be the main scission-inducing cue. Our finding is consistent with previous research that showed that afterimage formation is influenced by complex perceptual processes, and is not just dependent on physical stimulus qualities (i.e., luminance edges)^17–19^.

Consequently, our results suggest that a color diffusion-based account of afterimage filling-in is insufficient. In order to verify that a pure color diffusion-based mechanism cannot produce our data, we have performed model simulations on our stimuli (not shown) with a previously designed computational afterimage model based on color diffusion^6^ (and not scission processes). This model was unable to reproduce our data, suggesting that color diffusion alone is not able to correctly reproduce afterimage completion. Interestingly, this model has previously also been found to be unable to explain why a boundary placed completely *within* the area central gray patch is unable to stop filling-in of the area it surrounded^6^. According to the model the boundary should have stopped the diffusion of the color. However, an explanation based on scission is able explain these findings. Because the introduced boundary is surrounded by the same color of the central patch (gray) on both sides, it will be interpreted as a wire-figure lying in a depth-plane above the central patch, and it will not be considered a boundary of part of the central patch. Therefore, the visual system performed the completion process, ignoring the introduced boundary.

Previous research has suggested that contrast induction may also influence afterimage perception in the central patch, by inducing a color in the central patch opposite to that of the star points that are *not* outlined^5^. Potentially, this effect may strengthen afterimage completion when the induced color is similar to the afterimage color, and weaken it when it is not. This may provide an alternative explanation to the results of experiment 2. However, this color induction is a comparatively weaker effect^5^. Furthermore, color contrast is strongest at equiluminance^20^, while our effect peaks below equiluminance in experiment 1 (as well as in a pilot experiment), consistent with transparency. We also confirmed that a model based on the assumption that afterimage completion is strongest at equiluminance was 22 times less likely to explain the data in experiment 1, than a model incorporating transparency rules. Finally, in experiment 3, we found a strong correlation between afterimage completion of experiment 1 and 2, and transparency perception, which would not be predicted based on color contrast. These results suggest that, even though color contrast may modulate our effects, transparency cues are likely a more important determinant for visual completion.

**Figure 4:**
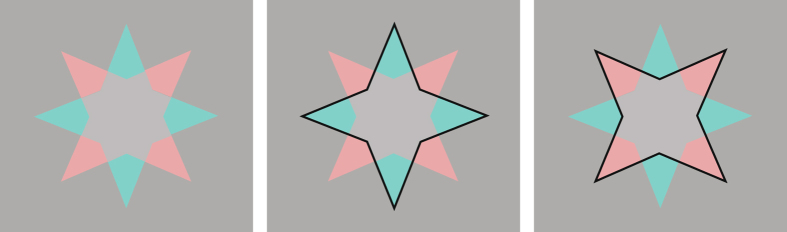
Examples of completion of the uncolored patches in non-afterimage stimuli. (Left) Staring at this image for a while (~10 s) causes the scene to decompose into a red and green star. The central grey area is then perceived as having both a red and green color. When one of the stars disappears due to adaptation, the other star is clearly perceived as having one continuous color. (Middle, and Right) Presenting a boundary around one of the stars may speed up the process of image segmentation. Note, that this stimulus is shown close to equiluminance to allow for the disappearance of one of the stars due to adaptation, but the color completion will occur even when the background is white (as in the original van Lier *et al*. figure).

Overall, our data argue for an alternative interpretation of afterimage data. Where previously, afterimage perception was often discussed in terms of lingering effects of adaptation, our data clearly show that afterimages are processed in very similar ways to “real” images (consistent with previous arguments^21–23^), and thus are due to an active ‘constructive’ process. If this equivalence between afterimages and ‘real’ images is true, then our findings should extend to such ‘real’ images. And if surface color perception is dependent on scission, we should be able to show that uncolored surfaces can become perceptually colored because of scission. Indeed, we find that a non-afterimage version of the star also displays completion of the gray area (Fig. 4), and additionally induces a concomitant change in the perception of the star’s points, which will appear transparent. This last observation is not predicted based on a simple filling-in account without taking into account scission. These results also show that the introduction of an artificial boundary, outlining one of the stars, is not a necessary requirement for completion to take place, although it does make completion clearer.

In conclusion, our findings suggest that during the perception of transparent surfaces, the color of both the bottom and top surface are represented explicitly, and the afterimage (as well as the color completion) is a reflection of that layered representation. This representation can be driven by transparency cues, and may be biased (but is not driven) by the introduction of visual boundaries.

## Methods

### Participants

We aimed to recruit a similar number of participants as in previous studies (15–20; see e.g. ref. 6). A power analysis based on previous experiments (ref. 6; figure 6, compare ‘middle’, in NB-NB vs NB-B, assumed correlation 0.5) suggests an estimated required sample size of 3 participants. Our experiments were performed in accordance with the Declaration of Helsinki, and approved by the Monash University Research Ethics Committee. The data were collected in accordance with ethical guidelines pertaining to the use of human subjects. All participants provided informed consent.

In experiment 1, we recruited 15 participants, of which 3 were excluded based on the predetermined exclusion that there should be no more than 2/3 of the trials without an afterimage, one of these participants also failed to adjust the stimuli to subjective equiluminance. (Note, due to feedback from a reviewer on a previous version of the experiment, we have also analyzed the data without excluding any participants; the results are very similar, and no results change in terms of whether they are significant or not.) Of the remaining 12 participants 3 were male and 9 female, with an mean age of (M ± std): 20.5 ± 2.4 y. All participants reported normal visual acuity and color perception.

In experiment 2, twenty-one participants participated; 12 had participated in Experiment 1. One participant was excluded because of a failure to adjust the stimulus in the equiluminance settings (same as in experiment 1). The remaining participants were 5 males and 15 females, average ± standard deviation age was 20.7 ± 2.4 y. None of the participants met the predetermined exclusion criterion of >120 trials without an afterimage.

In experiment 3, seventeen participants participated. Twelve of the 17 participants had participated in experiment 1, and all 17 in experiment 2. One participant was excluded because of a failure to adjust the stimulus in the equiluminance settings (same as in experiment 1), resulting in 16 participants with an overall mean age of (M ± std): 20.3 ± 2.2 y (12 female, 4 male).

### Design and Procedure

The stimuli were created in Matlab in conjunction with the Psychophysics Toolbox^24, 25^, and were presented on a 23” Tobii TX-300 screen (1920 × 1080 pixels, 60 Hz). Participants were seated in a dark room with their heads resting on a chin rest fixed at a distance of 70 cm from the monitor. Stimuli were presented at subjective equiluminance. A small black dot was provided as a fixation aid.

#### Subjective equiluminance

Prior to the experiments, participants took part in a brief session to determine their point of subjective equiluminance for red/green and yellow/blue stimuli. Participants were presented with a disk (4.3°) at fixation that flickered (26ms per color) between red and green (CIE(x,y) = 0.2269, 0.2967; Luminance (L) = 82.9 cd/m^2^), or blue and yellow (CIE(x,y) = 0.2618, 0.2912, L = 94.0 cd/m^2^), on a gray background (CIE(x,y) = 0.2474, 0.2613, L = 50.9 cd/m^2^) and adjusted the color of the red or blue disks until perceptual flicker disappeared. They did so 5 times consecutively for both color combinations. We took the average of the five settings for each combination to present in the subsequent experiments.

#### Stimuli Experiment 1

An eight-pointed star-shaped figure (see Fig. 1B) comprising of points that were alternating red (average settings: CIE(x,y) = 0.2851, 0.2615; Luminance (L) = 84.71 cd/m^2^) and green (CIE(x,y) = 0.2269, 0.2967; L = 82.9 cd/m^2^) or blue (average settings: CIE(x,y) = 0.2287, 0.2289; L = 93.0 cd/m^2^) and yellow (CIE(x,y) = 0.2618, 0.2912, L = 94.0 cd/m^2^) and a gray center patch that varied in luminance over trials (L = {0.1, 1.5, 8.3, 19.2, 34.1, 54.9, 83.7, 121.4, 169.0 cd/m^2^}, average CIE(x,y) = 0.2397, 0.2515). Stimuli were displayed on a white background (169.0 cd/m^2^), and were 4.3° wide and high.

#### Stimuli Experiment 2

The stimuli were identical to experiment 1, with the following changes. The center patch had a fixed gray value (CIE(x,y) = 0.2474, 0.2613, L = 50.9 cd/m^2^). The points were either red and green, or blue and yellow, or all the same color (all green, blue, red, or yellow). When the points had different colors, the stimulus was consistent with a transparent overlay of two stars with different colors. However, when all the points had the same color, no transparency was perceived.

#### Stimuli Experiment 3

All the stimuli of experiment 1 and 2.

#### Procedure Experiment 1

The adapting star was shown for 1.2 seconds, and was followed by a line that outlined the contours of the red points (plus center) or green points (plus center). This outline was displayed for 1 second before it was replaced by a mask (1.2 seconds, 256 × 256 random pixel array that covered the area of the star). Subjects were instructed to indicate whether (1) the afterimage consisted only of colored points, or (2) the entire outline was filled with one color, or (3) there was no afterimage.

We also performed a pilot experiment on 18 different individuals, with a stimulus shown for 4 s, followed by an outline shown for 1.2 seconds around one star, and followed by a second outline (1.2s) around the other star. Subjects then reported only on afterimage completion of the second outlined star. We report the statistics for this pilot experiment in the main text as well.

To test for the quadratic dependence on luminance we performed a linear-mixed model analysis, comparing a model with and without a quadratic dependence on luminance. Luminance was coded as 1–9 (different luminance levels). The model with a quadratic dependence was: ProportionCompletion ~ CenterLuminance + CenterLuminance^2 + (1|SubID). The model without the quadratic dependence did not include the CenterLuminance^2 term. Models were compared with the likelihood ratio test^26^.

We also compared two competing conceptual models to explain afterimage completion. One model is based on the transparency rules. This model assumes that completion (C) is highest (and constant) in the region consistent with transparency, and decreases on either side of this region. Specifically, this was modeled as:

1 $$C=\{\begin{array}{c}M-{s}_{1}({B}_{1}-L),{\rm{if}}\,L < {B}_{1}\,\\ \,M,{\rm{if}}\,{{\rm{B}}}_{1}\le {\rm{L}}\le {B}_{2}\,\\ M-{s}_{2}(L-{B}_{2}),{\rm{if}}\,L > {B}_{2}\end{array}$$

where M is the maximum proportion of completion, s_1_ and s_2_ are the slopes of the descending lines on either side of the transparent region. L is the luminance level (on log space), and B_1_ and B_2_ are the two luminance bounds to transparency (41.7 cd/m^2^ and 84 cd/m^2^, respectively).

The second model assumes that completion depends on low-level cues that predict that the proportion of completion is highest when the center and the points are equiluminant, and decreases on either side of this point. Therefore,

2 $$C=\{\begin{array}{c}M-{s}_{1}(E-L),{\rm{if}}\,L < E\,\\ \,M,{\rm{if}}\,L=E\\ M-{s}_{2}(L-E),{\rm{if}}\,L > E\end{array}$$

Symbols are as in the previous model. E is the point of equiluminance (E = B_2_ = 84 cd/m^2^).

In both models, M, s_1_ and s_2_ were free parameters. Models were fit with the fitnlm function in Matlab 2015b. Models were compared by calculating the Bayesian information criterion^10, 11^, and calculating posterior probabilities (“Schwarz weights”)^13^. We note that because the models have the same number of free parameters, these results will be equivalent using AIC or log-likelihood comparisons.

#### Procedures Experiment 2

The procedure was identical to that of experiment 1.

#### Procedures Experiment 3

All stimuli were presented to the participants 20 times and in random order, for 1 second. Participants indicated with a button press whether the stimulus appeared transparent to them or not. This allowed us to calculate the proportion of transparent percepts for each stimulus per participant. We then averaged the responses over participants (n = 16) for each stimulus (n = 9 in experiment 1, and n = 8 in experiment 2). These averages were then correlated to the average proportion of trials with afterimage completion for both Experiment 1 and 2 separately. This experiment was performed after experiment 1 and 2.
